# Expanding access to healthcare for people who use drugs and sex workers: hepatitis C elimination implications from a qualitative study of healthcare experiences in British Columbia, Canada

**DOI:** 10.1186/s12954-024-00991-2

**Published:** 2024-04-04

**Authors:** Nance E. Cunningham, Jessica Lamb, Amanda Staller, Mel Krajden, Robert S. Hogg, Angela Towle, Viviane Dias Lima, Kate Salters

**Affiliations:** 1 HIV/AIDS Drug Treatment Program, British Columbia Centre for Excellence in HIV/AIDS, St. Paul’s Hospital, 608–1081 Burrard Street, Vancouver, BC V6Z 1Y6 Canada; 2 Division of Infectious Diseases, Faculty of Medicine, University of British Columbia, 170-6371 Crescent Road, Vancouver, BC V6T 1Z2 Canada; 3 AIDS Network Kootenay Outreach and Support Society, 209a 16 Ave N, Cranbrook, BC V1C 5S8 Canada; 4 East Kootenays Network of People Who Use Drugs, 418-304 Street, Kimberley, BC V1A 3H4 Canada; 5 British Columbia Centre for Disease Control, 655 West 12th Avenue, Vancouver, BC V5Z 4R4 Canada; 6 Simon Fraser University, 8888 University Dr W, Burnaby, BC V5A 1S6 Canada

## Abstract

**Background:**

Hepatitis C virus (HCV) is a major health threat in Canada. In British Columbia (BC) province, 1.6% of the population had been exposed to HCV by 2012. Prevalence and incidence of HCV are very high in populations of people who use drugs (PWUD) and sex workers (SW), who may experience unique barriers to healthcare. Consequently, they are less likely to be treated for HCV. Overcoming these barriers is critical for HCV elimination. This research sought to explore the healthcare experiences of PWUD and SW and how these experiences impact their willingness to engage in healthcare in the future, including HCV care.

**Methods:**

Interpretive Description guided this qualitative study of healthcare experiences in BC, underpinned by the Health Stigma and Discrimination framework. The study team included people with living/lived experience of drug use, sex work, and HCV. Twenty-five participants completed in-depth semi-structured interviews on their previous healthcare and HCV-related experiences. Thematic analysis was used to identify common themes.

**Results:**

Three major themes were identified in our analysis. First, participants reported common experiences of delay and refusal of care by healthcare providers, with many negative healthcare encounters perceived as rooted in institutional culture reflecting societal stigma. Second, participants discussed their choice to engage in or avoid healthcare. Many avoided all but emergency care following negative experiences in any kind of healthcare. Third, participants described the roles of respect, stigma, dignity, fear, and trust in communication in healthcare relationships.

**Conclusions:**

Healthcare experiences shared by participants pointed to ways that better understanding and communication by healthcare providers could support positive change in healthcare encounters of PWUD and SW, who are at high risk of HCV infection. More positive healthcare encounters could lead to increased healthcare engagement which is essential for HCV elimination.

## Background

Canada has committed to eliminating hepatitis C virus (HCV) infection as a public health threat by 2030 [1]. Chronic HCV infection can progressively damage the liver, potentially resulting in cirrhosis and liver cancer. HCV had infected an estimated 1.6% of the population in British Columbia (BC), Canada by 2011–2012 [2].

People who currently use or formerly used drugs (PWUD) and with current or former work in the sex trade (sex workers, SW) have particularly high HCV incidence and prevalence [3, 4]. These two populations are not mutually exclusive, and the PWUD population in BC is difficult to define and estimate. Most figures for PWUD relate to a subset, people who inject drugs, PWID. A recent study estimated 65% of BC’s PWID will be exposed to HCV in their lifetime, and that the PWID population comprised 1.2 to 1.5% of British Columbians [5]. In BC at the end of 2015, 45% of people diagnosed with HCV were PWID, and recent research estimated that 80% of incidence was in PWID [4, 6]. A prospective cohort of PWID in the largest urban area of BC found HCV incidence of 3.1/100 person-years (PY) between 2006 and 2012, despite widespread availability of free harm reduction supplies [7]. HCV can be transmitted by non-injection drug use as well, although less efficiently [8].

Estimating the SW population in BC is speculative, so prevalence is uncertain [9]. However, two recent studies in Vancouver measured HCV antibodies in cohorts which included SW. Goldenberg et al. found that 44% of 759 SW in their Vancouver study had been exposed to HCV [3]. Incidence between 2010 and 2014 in this SW cohort was 3.8/100 PY. Incidence was elevated in participants using non-injection crack (6.3/100 PY), and 23.3/100 PY for participants using injection drugs. Shannon et al. found that among 3074 youth who injected drugs in Vancouver, 44% of those that those who did not work in the sex trade had evidence of HCV infection, which rose to 60% of youth involved in survival sex work [10].

The Treatment as Prevention (TasP) paradigm, initially a strategy to reduce HIV incidence in BC through early treatment of all eligible persons, can also apply to HCV elimination [11–13]. HIV and HCV differ in two ways relevant to TasP: curability and reinfection. As HIV is a lifelong infection, HIV TasP focuses on reducing transmission through case-finding and rapidly supressing and maintaining supressed viral load [14]. HCV TasP concentrates on case-finding, treatment, and follow-up as needed to reduce the risk of or promptly treat reinfection [15]. The microelimination approach complements TasP by structuring the response to ongoing incidence, identifying potential transmission networks and offering testing, treatment, and prevention simultaneously to all people in them [16–19].

BC took a critical step in operationalising HCV TasP in 2018 by removing disease-stage eligibility for care covered by the province’s universal medical services plan. This publicly funded HCV care covers antibody and RNA testing, diagnostic investigations, direct-acting antiviral (DAA) and other needed treatment, and follow-up at no cost to patients [20, 21]. Expanding eligibility resulted in increased treatment uptake but not equitable access [22, 23]. Treatment uptake in high-incidence populations remained under 50% in BC in recent data [22].

Eliminating HCV as a public health threat requires greater healthcare engagement with PWUD and SW populations, who bear a disproportionate HCV burden [1, 24]. Simple and highly effective DAA treatment created a prospect for elimination although critical health system and service barriers are hindering access to HCV care in these high-incidence populations. Many PWUD and SW are disengaged from healthcare, with stigma often cited as the primary reason for reluctance to engage [25–27]. Understanding the barriers, including prominently stigma from healthcare workers, which these populations commonly face when seeking healthcare and how some of these populations’ members have overcome them provides an opportunity to promote access so that those at high risk of HCV can receive equitable care.

To this end, this research explores healthcare experiences and relationships of PWUD and SW, and how positive and negative experiences affect their willingness to engage in future healthcare, including HCV care.

## Methods

### Theory and methodology

Interpretive Description, a qualitative research approach for applied health research, guided this project [28]. The Interpretive Description methodology is suited to this research as it can incorporate professional knowledge and theoretical frameworks to guide interpretation toward pragmatic rather than theoretical understanding [28–30]. Therefore we designed interviews to elicit accounts of specific experiences, and other material contributing to the understanding of the participants’ experiences as they related to the accessibility of healthcare in general and for HCV specifically, to inform recommendations for increasing healthcare engagement.

**Fig. 1:**
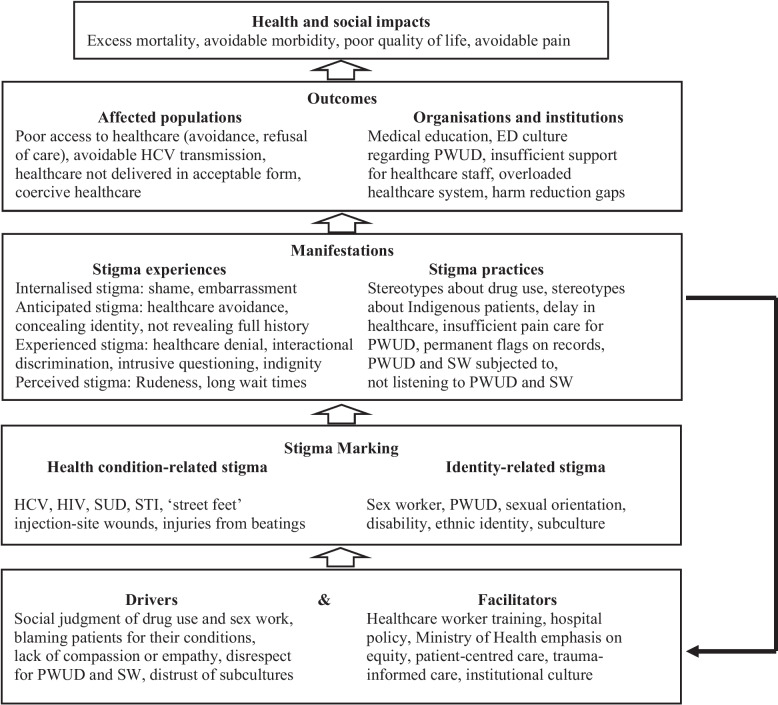
Findings from BC PWUD and SW in the health stigma and discrimination framework

The Health Stigma and Discrimination Framework (HSDF) proposed by Stangl and colleagues to link stigma and health outcomes is the theoretical framework we used [31]. (See Fig. 1) The HSDF builds on previous work on health-related stigma in the Goffman intellectual tradition [32–34]. As this framework is not specific to particular health or life conditions, nor a place or time, it can be used in applied research to trace the flow of antecedents of stigma through multiple steps and levels to their impact on individual and population health outcomes. The framework allows theory-based identification of potential intervention points. The framework includes ‘drivers’ which reinforce stigma but also ‘facilitators’ which can decrease stigma. The HSDF informed this study’s interview guide and a priori coding, and led us to focus on drivers leading to stigma manifestations and consequences and facilitators which can ameliorate stigma, rather than on the experiences of stigma as such.

Text in this figure was drawn directly from data and may include items not quoted. BC, British Columbia; ED, emergency department; HCV, hepatitis C virus; HIV, human immunodeficiency virus; PWUD, people who use drugs; STI, sexually transmitted infection; SUD, substance use disorder; SW, sex workers.

The research team included two persons with lived or living experience of key aspects of the populations studied. Methodological and subject matter experts filled out the remainder of the authorship team. A checklist consolidating qualitative criteria proposed by Tong and colleagues influenced the reporting processes [35]. Ethical approval for this research study was obtained through the Simon Fraser University Research Ethics Board, approval H20-02176.

### Sampling and data collection

The inclusion criteria for this study included informed consent, being at least 19 years of age, self-identification as someone who had past or present experience of drug use or work in the sex trade, and willingness to participate in interviews in English on their experiences in the BC healthcare system.

Sampling for this study was purposive. We sought potential participants between May 2021 and July 2022 using various strategies. First, research team members with experience of drug use, sex work, and HCV contacted participants in person at harm reduction sites in cities and towns in the rural parts of BC and through their personal networks and provided them information the study and contact information. Second, regional Drug User Groups, harm reduction, and supportive service organisations for PWUD and SW posted printed and electronic posters with the study’s recruitment text and contact information. These included the Northern BC Network of People Who Use Drugs, AIDS Network Kootenay Outreach and Support Society, and Harm Reduction Saves Lives. Third, in chain sampling interviewees could pass study information onwards to others in their social networks. Sampling was adaptive, to ensure participation of people with experience outside of the main metropolitan area of BC, as Metro Vancouver PWUD in BC are overrepresented in health research relating to drug use. We also prioritised SW, who have been underrepresented in HCV research. NC had no relationship with participants prior to the commencement of the study. JL and AS were acquainted with some of the participants.

Potential participants contacted the lead author by telephone or email to inquire about the study and receive a consent form. Consent forms were delivered to participants via their choice of email attachment, mobile telephone multimedia message, or on paper. Participants returned signed forms electronically or on paper, or informed the team that they could not return them. At this contact, an interview was scheduled for at least 24 h later. Participants who could not return a consent form gave verbal consent before the interview began. The investigators did not require identity documentation, allowing complete anonymity. No participants dropped out or declined to answer questions. All contact with potential and actual participants was virtual, due to COVID-19 pandemic restrictions, with the exception of participants who collected the honorarium in person, which was done outdoors. Interviews were recorded by Zoom® or GoToMeeting® videoconference software, with or without video depending on participant preference or equipment availability. NC initiated calls in a private room at a secure location, and JL joined some calls from a private room. Participants joined from a place of their choice.

Each participant took part in one semi-structured interview of 40 to 120 min and was compensated a minimum of CAD$30 per hour in cash or bank transfer for their time and contributions. The interview guide was pilot tested jointly by NC and JL, reviewed by VDL and KS, and twice revised. Interview questions evoked the quality of healthcare relationships and encounters, and factors that improved or detracted from these experiences.

### Analysis

Following the phases for rigorous thematic analysis as outlined by Nowell et al., NC transcribed interview recordings verbatim (deleting some filler words) and annotated them immediately following the interview [36]. NC wrote field notes in transcripts, a research journal, and in QSR NVivo® 12 software [37]. KS read transcripts; NC and JL, a community researcher, reviewed transcripts multiple times becoming familiar with the data. A priori codes had been posited from peer knowledge and theory (cf. Interview guide in Appendix 2). These codes were revised and further codes generated in a deductive–inductive iterative process. We sought themes related to a priori codes (e.g., healthcare avoidance) and the HSDF in a deductive process. Inductive analysis constructed themes (e.g., fear and trust in healthcare relationships) which emerged from the data through theory and researchers’ intuition from lived experience. Patterns and connections between experiences and actions (e.g., consequences of having drug use identified in medical care and refusal of care, building trust with healthcare providers and greater willingness to engage) were recorded in notes and memos as they became evident in the coding. We collated patterns from participants’ answers into themes. Proposed themes and sub-themes were reviewed, rearranged, renamed, and some eliminated during rounds of analysis and discussions between NC and KS, JL, and AS.

NC managed the study data including transcripts, field notes, versions of codebooks, and analytical memos in NVivo® and reflexive notes in a research journal. NC deidentified transcripts during transcription after which recordings were securely deleted. Deidentification concealed places, dates, other persons, work, and non-salient medications and health conditions. Participants did not comment or correct transcripts, but they could request a printed copy of their deidentified interview; two participants did.

The data collected satisfy the definition of meaning saturation [38]; however, the goal was not theoretical or thematic saturation. Following Interpretive Description, we considered sampling sufficient when the breadth of experiences, including geographical spread and diverse or contrasting cases, was appropriate to create knowledge to inform the practices relevant to this research [39, 40].

KS contributed to methodology choices and identifying a priori codes. NC, KS, and JL read transcripts. KS, VDL, NC, and JL developed, piloted, and revised the interview guide. NC and JL contributed to identifying a priori and emergent codes, coding, analysis, and interpretation. NC drafted the paper. All authors reviewed drafts and contributed to the interpretation. NC made the final selection of themes to be presented and examples to illustrate each theme.

## Results

### Sample

Twenty-five participants were interviewed, including 15 women, nine men; two participants used neutral pronouns, one of whom also used male pronouns. Of the 11 HCV infections discussed (including one case of reinfection and one of a close relative of a participant), six were cured and five were not treated. Participants brought up their status in five populations recognised as having elevated exposure to HCV: 24 participants spoke of their use of drugs (previous or current), two mentioned Indigenous identity, three men spoke of sex with men, 12 had previous or current sex work, and 12 had experience in correctional institutions. In addition, 11 participants mentioned mental health diagnoses and 11 experience of being unhoused.

### Themes

Participants described their experiences accessing healthcare, their willingness to engage in care, and the critical importance of communication by healthcare professionals in their experience. Their relationships, whether brief in a single encounter, or extended in a hospital stay or primary care attachment, were shaped by patterns of communication that healthcare workers may not be conscious of.

We present the findings in three major themes: (1) *“Other than, lesser than” Access to healthcare*, which collects data on whether or not participants received care; (2) *“It’s hard to reach out for help” Choices of healthcare avoidance or engagement*, in which the emphasis is on whether or not participants wanted care and under which conditions, and (3) *“Treat me like a human” Communication and relationships in healthcare,* in which participants describe qualities of verbal and non-verbal communication shaping their experience in healthcare and contributing to their willingness to seek healthcare*.* Some participants’ answers emphasised individual-level factors contributing to healthcare encounter quality, and others brought in institutional- or societal-level factors.

#### Theme 1: “Other than, lesser than” access to healthcare

This theme on participants’ access to healthcare collated cases when participants described their efforts to seek healthcare, their success or failure, and the impact of their perception of institutional culture. While almost all participants had some experiences of healthcare in BC which they labelled as good, the times when they did not receive such care stood out to them. Participants described common failures of healthcare, including delays or refusal of care for infection, illness, or injury, inadequate or absent pain management, and some counterexamples.

Notably, many experiences described involved multiple healthcare providers within the institutions providing care. In one example of delayed HCV care, a maternal health team diagnosed participant 13 (PWUD, SW) with chronic HCV but offered no counselling or path to treatment. *“… [T]he kids… I’ve been at risk over the years.”* She pursued HCV care through a low-threshold clinic after her primary care provider was slow to act when she became symptomatic:

*Participant 13: “I got frustrated when I wasn’t getting any results back … I had to go down on the [inner city] where a low-barrier hep C program is. I got my name on the list and that’s how I got treated.”*

While delay in HCV care was more common among participants than timely care, it should be noted that this sample was not representative of the PWUD and SW populations in BC. Nevertheless, it was particularly striking that so many of the participants did not have the first step in HCV care despite their high probability of exposure: knowing their HCV status.

HCV is rarely an emergency, but participants also spoke of being refused care in emergency departments (EDs) for serious conditions. Participants perceived the refusal of care to be related to their status as PWUD. The following quotes include one participant who worked in an ED and described the institutional culture regarding PWUD in EDs where they have worked.

Participant 4 (PWUD) was turned away from an ED with untreated bone fractures:

*Participant 4: “Yeah, broken [bones] for three weeks. And I didn’t [go to another hospital] because when I went … they did nothing to help me, and they dismissed me as a dirty drug user.”*

When she did seek healthcare again seen three weeks later, she was scheduled for surgery.

Participant 11 (PWUD, SW) was repeatedly refused adequate care in an ED over the course of several days as her health deteriorated, putting her long-term health at risk. She perceived that the delay in access to life-saving healthcare by multiple healthcare providers was due to her being identifiable as a PWUD:

*Participant 11: “… they didn’t run the proper tests that they should have if I was someone that wasn’t displaying signs of active addiction. So I ended up staying in the hospital for [a week and a half] with IVs connected and almost lost [an organ function] because of [the] infection.”*

A participant [all details withheld] who worked in an ED described the culture which led from people being perceived as drug-seeking to them being refused care.

*“If you are classified as dope-seeking or drug-seeking in Emerg, you are kiboshed. The quicker you get thrown out is the most rewarded behaviour. You are deemed an absolute powerhouse, not to be reckoned with, for throwing out the dope seeker. [laughs] You get props for that kind of stuff. Dope-seeking in Emerg is laughed at and not treated. And even more, people will boast that they caught it. … ‘We knew exactly was he was doing, didn’t get nothing out of us.’…Once you get labelled with drug-seeking, you’re done at Emerg. You’re not going to get treatment for a broken foot that day.”*

A phrase frequently used was *“lesser than”,* i.e., not being seen or treated equitably by healthcare professionals. Devaluing the health of PWUD could be fatal, as described by participant 12 (PWUD, SW). Participant 12 was waiting in an ED when another patient alerted medical staff that a third patient was showing diminished consciousness and other early signs of toxicity. The second patient suggested the nurse check his vital signs. Participant 12 heard an ED nurse falsely claim to have already checked him. The third patient went into the washroom and had a cardiac arrest with the door locked. Participant 12 saw a team responding to ‘code blue’, indicating he required resuscitation. She saw the team using a defibrillator, but she did not know whether he survived. She could not be sure if attentive staff could have averted the incident, but she witnessed the lack of urgency. She attributed the staff’s slow reaction to an institutional culture which dismissed the health and life of a PWUD:

*Participant 12: “They had the curtain, everything, shocking him and everything. The time they took to get that [washroom] door open because he was a dumb little addict is too long. It was about 20 minutes by the time they figured out how to get that door open. … And if she had done his vitals before, when the … lady asked her to?”*

Three further examples illustrate aspects of a particular kind of care refused in primary care and hospital settings. Pain management after injury or surgery could be insufficient or denied to participants who had been identified as PWUD. The first quotation depicts a typical example of a participant denied pain relief by healthcare providers who were more concerned with the danger of addiction than the intense pain. Another example describes healthcare providers deliberately cutting off pain medication, apparently for their own amusement. In each of these scenarios, the healthcare staff devalue the extreme pain suffered by the participants, creating an immediate problem and long-term mistrust.

Participant 8 (PWUD, SW) received only paracetamol with codeine in hospital after abdominal surgery, which she found to be inadequate to relieve pain. She was denied this and any further prescriptions once she left the hospital, leaving her in severe unrelieved pain. For her this was a stigmatising experience which she generalised into a profound reluctance to seek healthcare:

*Participant 8: “I hate them so much. It was that thing where you just feel so demeaned and so ‘other than’ and you’re just looking to get your needs met when you’re in pain. I had a 7-inch-long scar down the middle of my belly…. and they wouldn’t give me my medications…. So now when I’m sick or something’s going on… I’m like ‘No, they’re not going to help me anyway.”*

Participant 1 (PWUD, SW) described hospital staff deliberately exposing them to intense pain. Two hospital staff mocked up a morphine pump and dislodged their IV pain medication supply when transferring them to and from another care site. Participant 1 told how staff members ignored their distress:

*Participant 1: “They said, ‘Hey, when you’re with us you get this. You get that extra pump of morphine every five minutes.’… It wasn’t hooked up to anything. … I really got in my head about it for a long time afterward. I was like, ‘What would motivate someone to do that?’ … Well, prejudice against people who use drugs. … I started pouring sweat and … they were basically laughing at me. … It was like everyone was in on the joke.”*

It was alarming to Participant 1 that the medical staff had evidently planned together to deprive them of pain relief, implying that neglecting the pain of PWUD patients was condoned by institutional culture.

Participant 18 (PWUD) described multiple times healthcare providers refused to provide pain relief after injuries or invasive medical procedures, even years after he stopped taking any drugs but prescribed buprenorphine-naloxone. He perceived this to be due to the providers’ judgment that PWUD wanted the medicines for enjoyment, rather than for pain therapy:

*Participant 18: “It’s horrible…. It’s really unfair and not right that people should have to suffer in pain because [healthcare providers] think they’re getting something out of it by giving it to them. When I really am just getting relief. I don’t know. That’s a hard topic to talk about because I suffered so much.”*

Participants also described effective pain management. Participant 15 (PWUD, SW) was concerned about taking opioids when he had surgery within a year of stopping drug use. Concerned about relapsing, he tried to recover from surgery without asking for analgesia. He felt ashamed to ask for medication, but eventually he could not stand the pain. When he did ask, healthcare staff quickly administered morphine, saying, *“You don’t have to wait for it to be that bad. If you need help we can help you.”* Other participants reporting effective post-surgical pain care had their addiction specialist or family doctor communicate with the surgical team to plan the pain therapy.

#### Theme 2: “It’s hard to reach out for help” choices of healthcare avoidance or engagement

This theme gathered the variety of participants' desired and actual levels of engagement with the healthcare system. Participants fell into four categories, with some avoiding healthcare while acknowledging, and sometimes suffering, the risks of remaining untreated or treating themselves. These participants would only use emergency care, and some avoided even that. Others were able to retain a primary care provider who kept them engaged in healthcare even throughout years of problematic drug use, precarious housing, or work in the sex trade. They highly valued these long relationships. Between these endpoints were participants who relied on urgent-care or walk-in clinics for primary care. Some participants using walk-in clinics would prefer to have a regular family physician but were unable to find or retain one. Finally, others preferred walk-ins as they could choose how much of information to reveal. As seen in Theme 1, being identified as a PWUD could limit the care available, and some participants did not disclose their history. For these participants, BC’s patient-centred care policy did not provide them the care they desired. Centring the patient asks healthcare providers to look at the whole person, not just the health condition.

Some participants, including Participant 10 (PWUD, SW) found the “whole person” approach intrusive. “*I don’t need you to tell me what’s wrong with my life. … I just need some medical intervention.”* Rejecting such intrusion, Participant 10 told about treating an infection with prescription antibiotics on her own, and asserted that she would have sacrificed the limb to avoid going to a hospital where she expected to face stigma from healthcare providers:

*Participant 10: “I had an abscess once in an injection site. No way. I probably would have lost that arm before I would have gone into a hospital and said, like, ‘I’ve been injecting drugs with a dirty needle.’ … I had access to antibiotics. I medicated myself. ”*

Participant 13 refused to go with an ambulance whose crew tried to bring her to an ED after she escaped a murder attempt with injuries. She adamantly refused further treatment because she had been poorly treated in the past.

*Participant 13 (PWUD, SW): “I was covered in blood, … and I would* not *let them take me to the hospital. …I would have felt like I got raped over again, you know what I mean? The way how I’ve been treated in the past. I was not going to fucking put myself in a situation like that again.”*

In a case of a well-engaged person, Participant 9 (PWUD) attributed her consistent seeking of healthcare to good experiences in her youth. She was able to maintain a connection to care despite long periods of uncontrolled drug use and other challenging situations. *“When I was in addiction, as soon as I noticed anything, in I went.”* She attributed her survival to her strong engagement, as she rapidly sought treatment for a life-threatening antibiotic-resistant soft-tissue infection and received therapy promptly.

Participants described times when they were conflicted as they thought the correct thing to do was to seek care but they did not. These participants chose to treat their own medical conditions or go without care rather than seeking care from EDs or urgent care clinics like they ‘should’. Participant 17 (PWUD) described in detail how he used household tools to set his own broken finger rather than seek professional care. Participant 5 (PWUD) ended up hospitalised with an overdose after treating herself with medicines from a trusted friend. Participant 24 (PWUD) frequently injured himself at his job, and treated himself when he could. He described a cut which bled for four hours while he tried to glue it shut. *“I know I should go for stitches, but if I can crazy-glue’em, that’s where I’m at. If I have a broken toe or hands and shit, I just don’t go…. Oh yea, yea, I know.”*

Participants also changed their engagement in care. Participant 22 (PWUD) knew he had HCV but his primary care providers did not engage him on it so he *“just set it aside”.* After family and friends had good experiences with DAA therapy he sought treatment. *“I might as well give it a chance and not let [HCV] take too much of my health away. Before it’s too late…”.*

#### Theme 3: “Treat me like a human” communication and relationships in healthcare: Participants’ perceptions of the roles of respect, dignity, stigma, trust, and fear

This theme of communication and relationships in healthcare examines how the relational aspects of respect, dignity, stigma, and trust, were enacted or conveyed, and the effect of fear on communication between participants and healthcare providers. While most healthcare interactions explicated in the two themes above involved two-way communication, the participants focused their descriptions on other aspects. In this theme we look more closely participants’ perceptions of the effects of verbal and non-verbal communications.

Contrasting descriptions of attentive and dismissive one-on-one communication with a healthcare provider are seen in subsequent quotations. Participant 6 (PWUD, SW) described how verbal and non-verbal communication made a first encounter with a new family physician positive:

*Participant 6: “The first time I met him, he sat down and we discussed like all of my health concerns for an hour. And he sat there at my level and actually like he listened to me and explained everything in his perspective, and just, I felt really validated.”*

Participant 4 (PWUD), in contrast, spoke of encountering dismissive attitudes in healthcare settings where she thought more attentive healthcare providers should pick up non-verbal communication from patients who were not ready to communicate fully. In her experience, fear prevented her from saying what she needed to healthcare providers.

*Participant 4: “… people get really dismissed in a medical setting because the doctor knows best, and that’s it. So they’re not really listening to what you are saying. Or they’re not really listening to the things you’re not saying, which is: ‘I’m scared. I’m terrified. This is too much information for me to take in all at one time. Slow down.’ We don’t say those things.”*

Participant 23 (PWUD), who had untreated HCV, spoke of not being able to get the better of his fear when encountering healthcare providers during a drug-using phase of his life, preventing him from communicating the extent of his drug use. *“Yeah, in active addiction, probably wasn’t the most honest guy, you know. I was always fearful.”* This experience was echoed by Participant 22 (PWUD) who described the dynamic of active PWUD who *“are in protection mode all the time. It’s a learned behaviour. Trust, vulnerability, are off the table.”*

Participant 10 (PWUD, SW) had a long-standing relationship with a family physician who retired before DAA eligibility expanded to include Participant 10. She did not have enough trust in healthcare providers to speak to a new physician about HCV:

*Participant 10: “I don’t really know in what context I would bring it up with someone who doesn’t already know my history. I feel like it would make me extremely vulnerable…. Do I want to disclose that to a doctor I don’t know? Like, is he going to ask me questions about my past? Right now, I live in a very small community, right?”*

Participant 4 (PWUD) noted that she needed to have the courage to build trust with her healthcare provider and tell the truth about her drug-use history and be honest about her fear. She described how her physician showed he did not judge her and recognised her efforts, saying, *“Look, you know, these things happen. And you know you’re changing that around now….”* He gained further trust by asking if she would try things, contrary to what she had feared. She had expected him to force treatments on her.

Participant 2 (PWUD) pointed out that trust needed to be established on both sides. Healthcare providers frequently inquired about drug use more than 10 years after she ceased taking drugs. *“They always just assume that you still could be using and just not saying anything, right?”* This perceived mistrust detracted from her healthcare relationship.

Participants 10 (PWUD, SW) and 4 (PWUD) were among those who spoke about how communication about issues outside the ones the participant wished to raise could be perceived as judgmental and stigmatising. Participants tried to keep the discussion away from their history of drug use or sex work, and on the medical complaint they came for. *Participant 10:* “*It’s just it’s hard to reach out for help when you’re going to be stigmatised.”*

*Participant 4: “When you go in so broken … if they don’t handle [your history] well … you start feeling really embarrassed and shameful. So, you already got enough of that, trying to get out — even [*>*10 years] in sobriety — you already have enough of that to last a lifetime. You don’t need that from your healthcare professionals.”*

Respect can be expressed in verbal and nonverbal communication as well as actions. Participants found it important to communicate explicitly to establish a respectful relationship and recognition of their dignity. Participant 5 used a phrase that came out in many interviews: the wish to be spoken to and treated “like a human”. *“[They assume I have no education.] They won’t talk to me like I’m a human, really. Oh yeah, it’s awful.”*

Nonverbal communication was particularly important in whether people felt they could maintain their dignity Participant 11 (PWUD, SW) contrasted her perceptions of lacking dignity when she was laughed at to her later experiences:

*Participant 11: “I went into the washroom and used while being in the ER. And I had ... a small seizure… and the security were coming in, they started laughing at me. I was then put into a room with restraints … I was treated very poorly and with no dignity. Like, I felt like the scum of the earth. And I can definitely tell I was treated like that because I was in active addiction, because I’ve gone to the hospital after that while being clean and been treated totally different. Like, with morals, compassion, empathy. And I did not have that experience before that.”*

Participant 20 (PWUD, SW) maintained a strong relationship with a primary care provider during periods of drug use and sex work. One night she needed emergency care. A nurse’s comment had a near-fatal result and left an indelible memory:

*Participant 20: “I had an infection in my arm because of intravenous using and the [triage nurse] that was admitting me actually said, ‘Well it’s your own damn fault.’ … If I could’ve stopped, I would’ve stopped. … I was so filled with shame and guilt, I attempted suicide that night after I left the hospital. I’ll never forget her saying that to me.”*

Participant 19 (PWUD) was one of the participants who appreciated a healthcare provider drawing diagrams about their care for them in a combination of verbal and non-verbal communication:

*Participant 19: “She explained how everything was going to go… drew out diagrams for me … ‘this is what this is, and this is what that is.’ … Like she explained everything and what the [drugs] would do. It just– that really is reassuring. And you’re knowing what your medical journey is. It’s being totally explained to you, instead of living in the dark.”*

Participant 4 (PWUD) gave another positive account of an individual healthcare provider countering the effect of previous experiences. Her doctor asked her why she had avoided all healthcare for 10 years. After hearing of the times when she experienced indignity in healthcare, he explicitly took a position: *“[He said,] ‘I’m so sorry, you should never have been treated like that…. There’s no way that should have happened.’”.*

## Discussion

This study illustrated a wide range of healthcare experiences of PWUD and SW in BC. Negative experiences outweighed positive ones in participants’ recall. Low healthcare engagement among PWUD and SW has been shown in extensively in research, but most studies concentrate on healthcare avoidance on the part of PWUD and SW during active use and work, though there are exceptions [27, 41–44]. Our findings showed diminished access to healthcare through both participants’ avoidance of care and providers’ refusal to give care. Participants also reported the effects of negative experiences lasting for many years after drug use or sex work had ceased.

It has long been recognised that stigma detracts from many aspects of healthcare for people and populations that are labelled and devalued by healthcare professionals, reflecting general attitudes in their society [32, 34, 45–50]. Many negative experiences depicted in this study fell in the category of stigma manifestations, in terms of the HSDF. Negative experiences were traceable to the HSDF’s drivers of stigma, including lack of respect for PWUD and SW patients, lack of appropriate training, and institutional culture allowing inequitable treatment of PWUD and SW. PWUD and SW generalised their negative experiences, resulting in low seeking and uptake of care. Each participant could also recall healthcare experiences meeting BC Ministry of Health standards, i.e., quality, appropriate, and timely health services [51, 52]. Participants appreciated listening, trust, understanding, encouragement, respect, empathy, and compassion. Regarding the HSDF, these are the results of facilitators such as healthcare worker training, trauma-informed care, nonjudgmental institutional culture, and positive individual attitudes. Figure 1 shows the Health Stigma and Discrimination Framework with examples from this study [31].

Given the many efforts over decades to reduce stigma in healthcare, the findings of severe and long-lasting effects of stigma shown in detail in our findings are all the more troubling. Our results add to prior studies’ findings that the issue of stigma in healthcare was a high and consistent priority for PWUD and SW [53–56]. As other studies which explore patient experience as a PWUD, SW, or person with HCV we found current and former PWUD and SW populations presenting multiple reasons for low healthcare engagement, many at least partially credibly associated with stigma: experiences of dismissive attitude, intrusive questioning, blaming and other types of poor communication, delays in care, inadequate or inappropriate care, and withholding of care directly or indirectly reduced access to emergency, acute, and primary healthcare for participants [43, 44, 50, 57, 58].

Our findings offer positive and negative examples of how verbal and nonverbal communication affected healthcare relationships. Trust is recognised as an important aspect of healthcare [59–62]. Healthcare staff who spoke rudely, blamed participants for their own health issues, laughed at participants, asked questions not related to the medical intervention, lectured participants about their life or past as a PWUD or SW created distrust and reluctance to engage in healthcare. Clinicians who sat at the participants’ level, spoke empathetically when learning of participants’ history of negative experiences in healthcare, apologised for their institution, fully informed participants often by explaining processes with diagrams, shared decision-making, spoke nonjudgmentally about their past, and most importantly, listened respectfully could build trust. Explicitly addressing past stigma and adverse healthcare experiences, and demonstrating respect also built trust and dispelled fear. Participants in ongoing nonjudgmental healthcare relationships appreciated providers’ questions about past experiences in healthcare.

In literature on stigma in healthcare, fear is presented as felt by the more powerful party in an interaction, as a driver of stigma [31, 34, 63]. This study’s results can alert healthcare providers to the likelihood of fear being felt by patients with a history as PWUD or SW, especially in early visits with a new provider. Fear in our data was not only fear of anticipated stigma, but a generalised fear which inhibited participants’ ability to communicate with healthcare providers.

Provider-initiated HCV care was remarkably low. Delay or refusal of treatment is contrary to a TasP approach. The lack of care described by participants contributes to the expansion of the HCV epidemic as long as transmission of HCV remains high in populations with active drug use and sex work [3, 4]. The first step in HCV care is diagnostic testing, and since 1997 Canadian guidelines have consistently recommended tests for people who inject drugs and MSM [64]. However, we found that many participants did not know their HCV status, despite falling within testing recommendations. Of those who tested positive for HCV RNA, it was common for them to find care on their own initiative, or not seek care rather than having diagnosis and treatment or referral offered, per guidelines, by primary healthcare providers [65–67].

Changes in communication in ED have great potential as the ED is the only contact with the healthcare system for many PWUD and SW [42]. Study findings of the common occurrence of negative experiences in EDs suggest that more deliberate and respectful communication and efforts to build trust in emergency settings could be a step toward drawing people who avoid regular healthcare back into the primary care system.

Limitations of this study included the requirement to conduct interviews remotely due to Ethics Board requirements during COVID-19 restrictions, which biased the sample towards people in more stable situations which may be atypical for current PWUD and SW. This bias was mitigated by adaptively recruiting participants with living experience of drug use and sex work, and asking participants about past experiences. Another limitation was using a single main coder, increasing the risk of systematic personal bias. This limitation was mitigated by the co-review of transcripts and coding by JL, a research team member with lived and living experience of the conditions of interest. NC not being a member of the communities of interest was another limitation. This limitation was mitigated by having two team members with lived and living experience of HCV, drug use, and sex work. The ability to explore experiential issues such as engagement with sex work shaping PWUD experiences was limited by the choice of Interpretive Description as an approach, which directed attention away from deeper understanding of the of experiences of stigma, and toward implications for healthcare practice. A strength of this research was that it included experiences across the province, in contrast to the majority of research with PWUD and SW in BC concentrating on Vancouver’s metropolitan area or Downtown East Side, which has been described as one of the most heavily researched populations in the world [68]. Another strength is the inclusion of people who were known to have a high probability of exposure to HCV whether or not they had been tested, thus capturing more of the experiences of people who avoid healthcare and do not know their HCV status.

## Conclusions

Our study builds on previous evidence that healthcare engagement in PWUD and SW is low, and that stigma and other negative experiences decrease willingness to seek or accept healthcare. Low healthcare engagement will slow HCV elimination, as scale-up of HCV TasP and implementation of microelimination depend on a large proportion of people willing to engage in offered HCV testing and treatment.

In this study, collecting data on positive and negative experiences enabled us to identify potential points and means to support positive change in healthcare encounters of two high HCV-incidence populations critical to the success of elimination. While few healthcare providers deliberately undertreat, reject, or stigmatise their patients, providers should understand that many of their patients with histories of drug use or sex work have experienced stigma or inadequate treatment when seeking healthcare. Such negative experiences may have become generalised in PWUD and SW attitudes to all healthcare providers, creating fear of rejection, stigma, coercion, or refusal to provide adequate care. Healthcare providers can actively work to reduce the effects of negative healthcare experiences once they are aware of patients’ history and its long-term effects. Inquiring about past experiences, being aware of the tension between fear and trust, being explicit about accepting patients’ past without judgment and respecting their efforts to improve their health are all ways that healthcare providers can support patients with a history of drug use or sex work.

## Data Availability

Not applicable.

## Appendix 1

**Table:**

Codes, sub-codes	Description
Healthcare denial	Encounters in which people did not receive needed care Each should also be coded with Stigma, as an example of an outcome. Does not include examples where care that a participant had not sought is not given, e.g., participant suspected of drug-seeking but not offered resources or treatment substance-use disorder
Treatment delay	Examples when someone did not receive timely treatment, e.g., not suggesting DAA treatment when guidelines indicate they should be treated, not fully investigating conditions, injuries, etc. even if participant is able to get treatment by continuing to seek from other sources
Inadequate pain management	Encounters when post-surgical pain, or pain from an injury is not adequately treated and when e.g., invasive outpatient procedures are made without adequate treatment for pain
Failure to provide care	Encounters when the participant was turned away from care, and was not able to receive care within a reasonable period of time, e.g., people with broken bones turned away from emergency departments
Healthcare Engagement	General comments about healthcare experiences, and aggregation of more specific codes for healthcare experiences. Includes participants’ reluctance to seek healthcare, or willingness to engage in care. Most of these will additionally be coded for Valence
Avoidance	Instance of reluctance to get healthcare, including what thoughts were involved
Consistent care	Instances of pts maintaining continuity of care despite adversity, e.g., people who retain family doctor through uncontrolled drug use
Communication	Experiences in healthcare which depend on communication, verbal and nonverbal, between anyone in healthcare and the participant, in either direction. Includes showing empathy, lacking rapport, showing respect, expressing stigmatizing ideas, etc. Does not include counselling
Stigma and discrimination	Stigma, including experiences that the interviewee calls stigma, discrimination, shame. In academic terms, includes patient’s perceptions and experience of stigma (whether experienced, internalized, perceived, or anticipated), and antecedents of stigma (*marking* (e.g., as sex worker, substance user, street involved, ethnicity, mental health), *drivers* (e.g., judgment, fear, devaluing, whether mentioned or perceived by patients, or inferred from description), *facilitators* (e.g., attitudes expressed or inferred, e.g., trauma-informed service, policy, norms) and *practices* of stigma (e.g., asking irrelevant questions, dismissing concerns, blaming patient for condition), and *outcomes* (e.g., rationing of treatment, refusal of care (Note: these are double coded with a dedicated code, and additionally coded as ‘Negative’)
Fear and trust	Trust, distrust, mistrust, whether explicit or implicit, by healthcare professionals or participants. Also note that a lack of trust is often manifested in participants' fear of approaching a healthcare professional
Respect and dignity	Mention or examples of respect and disrespect, including respecting or disapproving of a person’s choice, situation, or condition. Do not use for judgment—judgment is of person, disapproval is of the choice or action. Code judgment under stigma
Valence	Cross-coding good/positive, bad/negative, or mixed/ambiguous/neutral in any other node
Ambiguous	Experiences, examples, quotes which are neutral, both good and bad, mixed, ambiguous, or neutral
Negative	Negative experiences, examples, quotes about what is bad etc.
Positive	Positive experiences, examples, quotes about what is good, etc.

## Appendix 2: Interview guide

### Interview guide for hepatitis C priority population healthcare experiences

Revised 9 Nov 2021

Thanks for this call. In this interview, we want to understand your experiences in healthcare system in BC. The ultimate goal of this project is to improve the quality of hep C care experience in BC. I am Nance Cunningham, and this is part of my PhD research. I am Jessica Lamb, *[Jess introduces herself].* You have chosen to talk with us for 30/60 min, but you can change your mind at any time, to speak for longer or shorter.

This interview is about your experience in healthcare, including what you have witnessed and felt. We/I don’t need to know your medical conditions, only about your view of your healthcare experience. What you tell us/me will remain anonymous, unless you have chosen to use your own name. We may quote you with the name you have chosen. The interview as a whole remains confidential, and can be read only by the research team. Only quotations of a few sentences may be published.

If you don’t want to answer a question, that’s no problem, just ask to go on to the next question. If you want to tell us/me something not asked, please do. Do you have any questions about the interview? [Answer any questions]

[As Jessica told you earlier], we/I will record and write out this interview to be sure of your exact words. I will start recording now. [Start recording] You can ask me to stop any time. Let us/me know if you need a break for any reason.

[*For those who have not been able to provide informed consent, ask for informed consent and pseudonym here:* Please state whether you understand this study, and consent to do this interview, and what name you would like to be used if we quote you.]

To start off, we/I’d like to ask you about your recent experiences in healthcare in BC. That is about in experiences in any kind of healthcare setting, it could be a hospital stay, a clinic visit, something that happened in an emergency room, with picking up a prescription, going to a lab, 911 call, anything like that. You can talk about what you experienced yourself, or what happened to someone else when you went with them.

**Table:**

Topic	Questions and prompts
Preferred HC	First we would like to know who you find your most reliable healthcare provider to be. Is there a place or person you turn to where you know you will get help? Probe: If you felt very sick tomorrow, who would you call or where would you go?
COVID	How has your healthcare changed during the COVID-19 pandemic? Probe: How is that for you?
Healthcare experiences	I’d like to start asking you about good and bad experiences you have had in healthcare settings (like in emergency departments, clinics, hospitals, pharmacies, when someone called 911, checking in with OAT, whatever). *Do you want to start with good or bad?*
Lower bound of experience	[Give this explanation only once, for either good or bad only, adjusting appropriately] I am going to ask you about a *bad experience* in healthcare. By bad experience, I mean the quality of your experience with the people there, compared to what it could have been. I don’t mean your worst health crisis, or a medical treatment that did not work. I mean an experience that would have been better if someone had treated you differently What is the worst experience you have had in a HC setting? Probes: [ask for further explanations according to context]
Upper bound of experience	What is the best experience you have had in a HC setting? Probes: [ask for further explanations according to context]
Draw out concepts	So from what you said, it seems like what made the experience good / bad was [list main points mentioned in good and bad experiences]. [For each, good and bad:] Is that a fair summary?
Intro to frequency:	Now I would like to ask you about how often you have good or bad experiences in the same kinds of healthcare settings, like clinics, pharmacies, and so on. Do you want to start with good or bad?
Frequency of bad experiences	What is the *most common* bad experience you have had in healthcare? Probe: [ask for further explanations according to context]
Frequency of good experiences	What is the *most common* good experience you have had in healthcare? Probes: [ask for further explanations according to context]
Draw out concepts (Ask separately for good and bad)	So from what you said, it seems like what made the experience good/bad was [list main points mentioned in good and then bad experiences]. [For each, good and bad:] Is that a fair summary?
What else is needed for optimal healthcare?	You have told us/me about many things that happened *in* healthcare settings. But sometimes, you didn’t / people don’t even go for healthcare. What else is needed for people [like you at that time] who mostly avoid healthcare to be able to get the health attention they need? Probes: [ask for further explanations according to context] What would have made it better for you?
Follow up topics	[If time:] Tell us/me more about [any relevant topic that came up]
Overall impression	How would you sum up your experience in healthcare in BC?
Conceptual framework	If you think about all the good and bad experiences you have had in the BC healthcare system, do you see them as different kinds? What kinds? Probe: What are the factors that make an experience good or bad?
Ideas for change	What are your expectations from the people who are involved in healthcare, including security, receptionist, doctors, nurses, anyone you might meet in a healthcare setting? Based on what you have told me, how would you like the bad encounters to change? What would have made them better How could care be improved for people who use drugs and/or sex workers? Probes: What could change so that they would not happen again? What could have been done better?
Priorities	What are your top health goals for yourself? What do you think your doctor’s top goals for you are? [Note for further research: Ask HCP this question as well] Probe: When you go for healthcare, what do you want from that appointment?
Message direct to healthcare provider	[Optional question] If you could say anything openly and directly to people who work in healthcare in BC, and you knew they would hear what you say, what would you say to them?
Hepatitis C history	Do you think HCV is a priority for PWUD/SW? Have you ever been tested for hepatitis C? Who would you prefer to see for hep C care? Probe: How often? Did you ever test positive?
If hepatitis C experienced	How do you find out you had hep C? Tell me about the diagnosis and treatment experience Probes: Are/were you comfortable asking your healthcare provider about hep C testing and care? Has this changed over time? Did you feel adequately supported? What counselling did you get before and after testing? Did you take a test after the end of treatment to confirm your cure? Was your care in getting treated/supported with HCV different to other types of care you received? How?
If not hepatitis C experienced	Is hep C a priority for you? (Or: Was it when you [were in the period when exposure was more likely]?) Probes: Learn about willingness to get testing, and testing experiences: Did you ever get tested? Did you feel adequately supported? Where would you go for a test? What counselling did you get before and after? How easy was it to find a place to get tested? How often would you get tested? Are/were you comfortable asking your healthcare provider about hep C testing and care? How has this changed over time? What would you need to feel comfortable getting tested / starting treatment?
Interview experience	If our positions were swapped on this interview, what would you do differently?
[Last 2 min] Recruitment	We would like to talk with more people. Do you know anyone who you think would like to speak with us? If so, please have them contact Jessica, or Nance at: 778 906-2382, or using the contact information you have

Thank you so much for talking with us/me today.

## Abbreviations

BC: British Columbia
ED: Emergency department
HCV: Hepatitis C
HIV: Human immunodeficiency virus
PWUD: Person (or people) who use (or used) drugs
RNA: Ribonucleic acid
STI: Sexually transmitted infection
SUD: Substance use disorder
SW: Sex worker
